# Enhancement of Biological and Pharmacological Properties of an Encapsulated Polyphenol: Curcumin

**DOI:** 10.3390/molecules26144244

**Published:** 2021-07-13

**Authors:** Bwalya Angel Witika, Pedzisai Anotida Makoni, Scott Kaba Matafwali, Larry Lawrence Mweetwa, Ginnethon Chaamba Shandele, Roderick Bryan Walker

**Affiliations:** 1 ApotheCom|A MEDiSTRAVA Company (Medical Division of Huntsworth), London WC2A 1AN, UK; bwalya.witika@apothecom.com; 2 Division of Pharmaceutics, Faculty of Pharmacy, Rhodes University, Makhanda 6140, South Africa; 3 Division of Pharmacology, Faculty of Pharmacy, Rhodes University, Makhanda 6140, South Africa; P.makoni@ru.ac.za; 4 Clinical Research Department, Faculty of Infectious and Tropical Diseases, LSHTM, London WC1E 7HT, UK; Scott.Matafwali@lshtm.ac.uk; 5 Department of Chemistry, Marine Biodiscovery Centre, University of Aberdeen, Aberdeen AB24 3FX, UK; larrymweetwa1@gmail.com; 6 Department of Biochemistry, Institute of Basic and Biomedical Sciences, Levy Mwanawasa Medical University, P.O. Box 33991, Lusaka 10101, Zambia; shandeleginnethon@yahoo.com

**Keywords:** encapsulation, polyphenols, curcumin, enhanced bioavailability, sustained release, targeted delivery, anti-inflammatory, anti-oxidant, anti-viral, cancer therapy

## Abstract

There is a dearth of natural remedies available for the treatment of an increasing number of diseases facing mankind. Natural products may provide an opportunity to produce formulations and therapeutic solutions to address this shortage. Curcumin (CUR), diferuloylmethane; I,7-bis-(4-hydroxy-3-methoxyphenyl)-1,6-heptadiene-3,5-dione is the major pigment in turmeric powder which has been reported to exhibit a number of health benefits including, antibacterial, antiviral, anti-cancer, anti-inflammatory and anti-oxidant properties. In this review, the authors attempt to highlight the biological and pharmacological properties of CUR in addition to emphasizing aspects relating to the biosynthesis, encapsulation and therapeutic effects of the compound. The information contained in this review was generated by considering published information in which evidence of enhanced biological and pharmacological properties of nano-encapsulated CUR was reported. CUR has contributed to a significant improvement in melanoma, breast, lung, gastro-intestinal, and genito-urinary cancer therapy. We highlight the impact of nano-encapsulated CUR for efficient inhibition of cell proliferation, even at low concentrations compared to the free CUR when considering anti-proliferation. Furthermore nano-encapsulated CUR exhibited bioactive properties, exerted cytotoxic and anti-oxidant effects by acting on endogenous and cholinergic anti-oxidant systems. CUR was reported to block Hepatitis C virus (HCV) entry into hepatic cells, inhibit MRSA proliferation, enhance wound healing and reduce bacterial load. Nano-encapsulated CUR has also shown bioactive properties when acting on antioxidant systems (endogenous and cholinergic). Future research is necessary and must focus on investigation of encapsulated CUR nano-particles in different models of human pathology.

## 1. Introduction 

Polyphenols are secondary metabolites found in all vascular plants and are ubiquitous and include diverse substances ranging from simple molecules to large complexes [1,2]. The compounds generally impart defensive properties against ultraviolet radiation or pathogen infections and are classified into groups depending on the number of phenol rings and structural elements that link the rings. Consequently, distinctions can be made between phenolic acids, flavonoids, stilbenes and lignans such as protocatechuic acid, coumaric acid, resveratrol and secoisolariciresinol, respectively [3], with other polyphenols including coumarin and condensed tannins based on structural attributes [2,4]. 

Polyphenols are one of the largest groups of natural compounds which exhibit a broad range of biological activities including anti-oxidant, anti-inflammatory, anti-allergy, anti-viral, anti-cancer, anti-microbial, anti-mutagenic and cardio-protective effects directly related to the diverse structural configuration of these materials [4]. Numerous research reports in which polyphenols are reported have focused mainly on determination of the molecular profile of the compound derived from various plant extracts [5,6], determination of the biological activity [7,8,9] and the subsequent development and optimization of unique extraction methods for different plant metabolites [10,11,12]. However, current research is focused on enhancing the bioavailability and pharmacological properties of phenolic compounds in the human body for the purposes of targeted and improved therapy [2,13,14]. 

**Figure 1:**
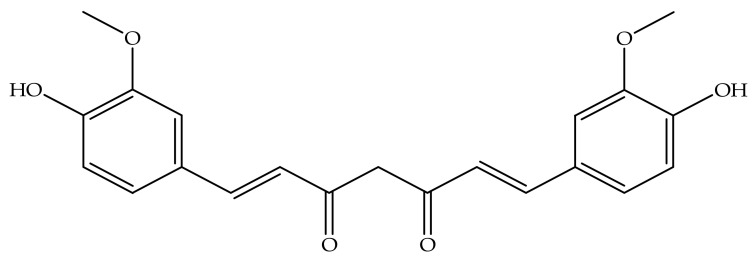
Chemical structure of CUR MW = 368.38 g/mol [26].

Bioactive compounds of natural origin are chemically unstable and are prone to oxidative degradation [15] which may adversely affect the integrity of the compound(s) and lead to the formation of free radicals [16]. Consequently, unpleasant tastes and/or odours are developed in the final product which have a negative consequence in respect of the shelf life, organoleptic properties, and consumer acceptability for and of the product [15,17]. Furthermore, the use of pure bioactive compounds in formulations for biological applications is limited due to undesirable characteristics such as low aqueous solubility, poor bioavailability and reduced stability in the presence of environmental stresses such as pH, high temperature and exposure to oxygen and light [15,18,19]. Therefore, to preserve quality or enhance the biological use of these compounds, encapsulation has been used as a practical approach for the purposes of protecting and delivering naturally derived bioactive molecules at the appropriate time to targeted sites [20]. Encapsulation may also be used to improve the solubility, minimise degradation, reduce toxicity and control the absorption and biological response that result when using polyphenols therapeutically [21,22]. To date, special attention has been given to specific bioactive molecules, such as curcumin (CUR) (Figure 1), due to the purported health benefits it imparts such as anti-oxidant, anti-inflammatory and anticancer effects [23,24,25]. However, CUR is poorly absorbed from the gastrointestinal tract (GIT) following oral administration due to low aqueous solubility and poor stability in GIT fluids, more specifically in alkaline pH conditions [22]. 

Several encapsulation techniques can be used for nutritional and therapeutic targeting of bioactive molecules [22], of which nano- and micro-capsules are considered the most functional and desirable [27]. In particular, micro-encapsulation using spray drying is the most common and widely used approach in the food and pharmaceutical industries [28]. Recently, nano-encapsulation has gained interest due to efficient encapsulation, enhanced stability, and a greater ability to control release of encapsulated material(s) [15,29,30]. Herein, some extraction methods and therapeutic properties of polyphenols are emphasised, in addition to evaluation of nano-encapsulation of CUR and associated therapeutic effects are reviewed with suggestions of future research direction. Articles in this review were extracted from electronic databases, viz. Scopus and PubMed, using the keyword curcumin. The title and abstract of articles were screened for relevance, to eliminate duplication and articles not relevant to this review. Consequently, the manuscript contains seminal (prior to 1976) and recent references where most (>70%) have been published within the last ten years (2011–2021) resulting in 171 references being included in this review. 

## 2. Origin, Extraction and Therapeutic Properties of Curcumin

CUR is a natural polyphenol derived from the plant *Curcuma longa Linn*, a member of the Zingiberaceae family [31]. The plant occurs naturally throughout many tropical and subtropical regions of the world and has been used in Ayurveda and traditional Chinese medicine for thousands of years to treat inflammatory disease(s) and bacterial infection(s) [32,33]. Since the first reported extraction and separation of CUR around 1815, novel, facile, efficient and sophisticated extraction procedures continue to be developed [26,34,35,36]. The most commonly reported extraction method for CUR from turmeric makes use of solvent extraction procedures followed by column chromatography. Different polar and non-polar organic solvents have been used in these extraction procedures, including hexane, ethyl acetate, acetone, methanol, and ethanol, which is the preferred solvent for extraction [26,34,37,38]. Natural deep eutectic solvents (NADES) are gaining popularity as novel green materials used in the food, cosmetic and pharmaceutical industry. NADES exhibit unique solvation properties with associated low toxicity and have been used in the extraction of CUR [35,39,40]. When compared to CUR extracted using conventional extraction solvents the CUR extracted using NADES exhibited greater total antioxidant capacity and higher CUR payload [35,40]. Apart from improving the solubility and stability of CUR [39] NADES extracts exhibited increased antioxidant activity possibly due to enhanced bioavailability resulting from the effect of NADES during antioxidant membrane transport [35]. Furthermore, Soxhlet [41], ultrasound [42], microwave [43], pH-zone-refining [44] and supercritical carbon dioxide [45] extraction methods of CUR have been reported.

CUR elicits several pharmacological activities including chemotherapeutic, anti-oxidant, anti-viral, anti-bacterial, and anti-inflammatory actions [46,47,48,49,50,51] which are further elucidated below.

### 2.1. Anti-Inflammatory Activity 

CUR has been reported to suppress acute and chronic inflammation by several mechanisms such as regulation of inflammatory responses by down-regulating the activity of cyclooxygenase-2 (COX-2), lipoxygenase and inducible nitric oxide synthase (iNOS) enzymes [52]. CUR also blocks the production of inflammatory cytokine tumour necrosis factor-alpha (TNF-a), interleukin (IL) -1, -2, -6, -8, and -12, monocyte chemo-attractant protein (MCP), migration inhibitory protein in addition to down-regulation of mitogen-activated and Janus kinases [46,53]. CUR has been shown to suppress NF-κB activation and proinflammatory gene expression by blocking phosphorylation of inhibitory factor I-kappa B kinase (IκB) which subsequently down-regulates COX-2 and iNOS expression, inhibiting the inflammatory process and consequent tumourigenesis [54,55]. Inhibition of inflammatory cytokines by CUR is achieved through several mechanisms. CUR was reported to regulate activation of transcription factors such as activating protein-1 (AP-1) and NF-κB in stimulated monocytes and alveolar macrophages, thereby blocking expression of cytokine gene expression [56]

Several studies have reported the effect of CUR in disease and recently, some studies have suggested that CUR may be useful for treating COVID-19 [57,58] due to immunomodulatory activity which reduces early inflammatory responses effectively improving the survival of severely ill hospitalised COVID-19 patients. Cytokines, particularly IL1β and IL6 are markedly elevated in severely ill COVID-19 patients and CUR may conceivably reduce the level of these cytokines [59,60]. 

CUR has also been shown to significantly reduce the levels of mediators of inflammation in pneumonia caused by *Klebsiella pneumonia* [61]. Several studies have also shown that CUR can alleviate the symptoms of inflammatory disorders such as rheumatoid arthritis and osteoarthritis [62,63,64].

### 2.2. Anti-Viral Activity

CUR exhibits antiviral properties and several studies have reported that CUR interrupts viral infection processes by different mechanisms, including prevention of viral entry, inhibition of replication, directly targeting viral proteins, blocking particle production and gene expression [65,66,67,68,69,70,71,72]. CUR has been reported to be effective against SARS-CoV in in vitro studies [68] and more recently it has also been postulated that it may have antiviral effects against SARS-CoV2, the virus that causes COVID-19 [57,58,73,74]. Wen et al. [68] reported that CUR inhibits SARS-CoV 3CL protease activity, which is vital for viral replication. Other studies have postulated that CUR can interfere with SAR-CoV2 entry and replication by interacting with the S protein and angiotensin converting enzyme-2 (ACE2) protein [73,74]. On this basis it has been postulated that CUR may directly intervene in SARS-CoV-2 entry and or replication and prevent infection. Furthermore CUR has been reported to also exert antiviral activity against SARS-COV-2 by activating the NRF2 pathway [58,75]. 

CUR has also been found to be therapeutic effective against other viruses such as the influenza A (IAV) and respiratory syncytial (RSV) viruses. CUR prevented replication in human nasal epithelial cells by inhibiting NF-κB and cyclooxygenase 2 signalling and also demonstrated in vitro inhibition of respiratory syncytial virus (RSV) by blocking attachment to host cells [67,69,76]. CUR inhibits IAV hemagglutinin, a homo-trimeric membrane glycoprotein, binding to host cell receptors [72] and has been shown to be effective in inhibiting and disrupting several steps in replication cycle of HIV including the manufacture of viral protease, integrase enzymes and HIV tat proteins [65,77,78]. 

CUR has been reported to exhibit antiviral effects to the hepatitis C [79], herpes simplex (HSV) [80] and Zika and Chikungunya viruses [81]. 

### 2.3. Anti-Cancer Activity

CUR possesses anti-cancer activity mediated through effects on several pathways involved in angiogenesis, mutagenesis, oncogene expression, cell cycle regulation, apoptosis, tumourigenesis and metastasis [82,83,84]. Furthermore CUR affects a variety of growth factor receptors and cell adhesion molecules involved in tumour growth [85,86]. Studies have demonstrated the inhibitory effect of cyclooxygenase (COX-2) by CUR in human colon cancer and human breast carcinoma cells has further inhibitory effects on ERK1 and ERK2, which are mitogen-activated protein kinases that facilitate cell proliferation and apoptosis signalling pathways [87,88,89]. CUR suppression of carcinogenesis in a host of cancer types has been demonstrated in vivo and in vitro including breast and prostate tumour models by exerting anti-proliferative and proapoptotic effects through activation of caspases, the release of cytochrome C and repression of cell survival factors via inhibition of the NFκB pathway ([90,91]). Further apoptotic effects of CUR include the inhibition of Akt/protein kinase B (PKB) phosphorylation in breast cancer cells [92]. CUR also has an effect in melanoma and glioblastoma based cancer which was observed via increased autophagy and decreased levels of mitophagy markers, in combination with inhibition of the PI3K-Akt/mTOR pathway when treating of glioblastoma cells [93,94].

### 2.4. Anti-Bacterial Properties 

Studies have revealed that CUR exhibits a broad spectrum of anti-bacterial properties by a range of mechanisms including inhibition of bacterial DNA replication by inhibiting the function of filamenting temperature-sensitive mutant Z which is necessary for bacterial cell division, alteration of gene expression, bacterial cell membrane disruption and a reduction in motility [50,95,96,97,98]. CUR can influence cell division and proliferation of bacteria by inhibiting polymerization of FtsZ proto-filaments and interrupting GTPase activity in the cytoskeleton of bacteria such as *Escherichia coli* and *Staphylococcus aureus* [99,100]. CUR exhibits apoptosis-like response in *Escherichia coli* [101]. CUR inhibits the growth of bacteria such as Streptococci, Staphylococci, Lactobacillus and inhibits strains of *Helicobacter pylori* strains in vitro by regulating expression of matrix metalloproteinase-3 (MMP3) and MMP9 [50,95]

### 2.5. Anti-Oxidant 

CUR possesses anti-oxidant properties and studies have revealed that CUR prevents the oxidation of important proteins such as haemoglobin [102,103]. CUR is an effective the scavenger reactive oxygen (ROS) and nitrogen species (RNS) which cause several diseases. CUR regulates the activity of GSH, catalase, and SOD enzymes which are active in neutralizing free radicals [49]. In addition, CUR was shown to inhibit ROS generating enzymes such as lipoxygenase, cyclooxygenase and xanthine hydrogenase and oxidase [104,105], and reduced the oxidative damage of DNA, providing protection against Parkinson’s disease, several autoimmune diseases, and aging [57,106]. Furthermore, CUR has been reported to protect against ochratoxin A-induced oxidative damage in the liver of rats [107] and improve the antioxidant capacity of poly(vinyl alcohol)-based films for potential application in food packaging [98]. 

## 3. Encapsulation and Evaluation of Therapeutic Benefits of CUR 

Encapsulation has long been used to overcome some of the challenges associated with improving effective drug delivery. In general the inclusion of polymers, lipids, and surfactants for the production of suitable carriers for medicinal and non-medicinal applications has been demonstrated and opportunities to produce technologies that exhibit advantages over conventional formulations including improved stability, enhanced and beneficial biodistribution profiles, altered drug release rates, lower immunotoxicity and with potential targeting of specific cells, exists [108]. Micro- and/or nano-encapsulation has been used to develop sustained/prolonged drug delivery technologies [30,109,110], minimise degradation in biological fluids [111], facilitate handling [112] reduce toxicity [109,113,114,115] and to improve the organoleptic properties [19,116,117] of products.

The process of encapsulation is a rapidly expanding area in the pharmaceutical sector as there is a need to develop technologies to protect and deliver different bioactive compounds [118]. Encapsulation is described as a process through which small core-material particles are converted into a capsule by surrounding and packing with protective shells [119]. Specifically, the core or coated material, is a pure compound or sometimes a combination of compounds with distinct chemical and physical properties.

The process of encapsulation involves a number of processes which includes wall formation around the core, testing to ensure leakage does not occur and ensuring exclusion of undesired materials such as unencapsulated drug [119].

**Figure 2:**
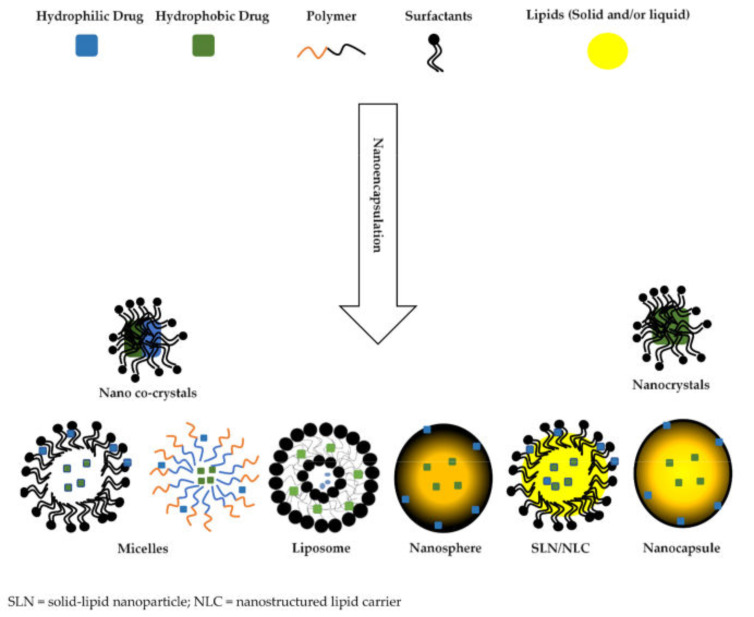
Schematic representation of various nano-encapsulated systems [108].

Various encapsulation approaches have been used to achieve a specific delivery profile or system and are summarised in Table 1, where the initial classification as physicochemical, chemical, and physical methods with associated sub-categories are listed. Furthermore, a schematic representation of nano-encapsulated systems is depicted in Figure 2.

### 3.1. Chemotherapy

To improve the applicability of CUR in cancer therapy the polyphenol was encapsulated in poly(lactic-*co*-glycolide) as nanoparticles, in the presence of poly(vinyl alcohol) and poly(l-lysine) stabilisers, using nano-precipitation [121]. The optimised CUR nano-formulation exhibited a two- and six-fold increase in cellular uptake using cisplatin resistant A2780CP ovarian and metastatic MDA-MB-231 breast cancer cells when compared to treatment with free CUR. In addition, the optimised nano-formulation exhibited improved anti-cancer potential in cell proliferation and clonogenic assays when compared to free CUR, which was attributed to enhanced apoptosis induced through use of the nano-carrier technology. In these studies, cells treated with equivalent amounts of DMSO (or empty), nanoparticles were used as controls for CUR and the optimised CUR nano-formulation, respectively [121]. CUR-encapsulated methoxy poly(ethylene glycol)/poly-ε-caprolactone di-block copolymeric micelles were prepared using a modified dialysis method and revealed that CUR-loaded nano-micelles increased the bioavailability of CUR due to an enhanced uptake of 2.95-fold greater than that of free CUR with comparative cytotoxic activity at equimolar concentrations. The IC_50_ values for pure CUR and CUR nano-micelles were 24.75 and 22.8 µM and 14.96 and 13.85 µM for MIA pancreatic cell lines, PANC-1 and MIA PaCa-2, respectively [122]. CUR was successfully encapsulated in monomethyl poly (ethylene glycol)-poly (ε-caprolactone)-poly (trimethylene carbonate) micelles achieved using a single-step solid dispersion method. In these studies, the free CUR and nano-micelle containing CUR suppressed the growth of CT26 colon carcinoma cells successfully, in vitro. Furthermore, the CUR nano-micelles were found to be more effective in suppressing tumour growth of subcutaneous CT26 colon carcinoma cells, in vivo through inhibition of tumour proliferation, angiogenesis, and increased apoptosis of the cells. Furthermore, growth medium without CUR and cells cultured in medium without treatment reagents were used as the control [123]. The anti-cancer activity of free and poly lactide-co-glycolide-co-polyacrylic acid nano-encapsulated CUR on PANC-1 cell lines was reported. This study revealed that free and nano-encapsulated CUR significantly increased apoptosis rates and decreased viability, migration and colony formation in the PANC-1 cell line with the nano-encapsulated CUR exerting a more profound apoptotic and anti-proliferative effect on the PANC-1 pancreatic cell line than the free CUR [124]. 

Nano-sponges (NS) are sponge-like nanometric sized materials which have been used to encapsulate a wide variety of drugs. NS have an ability to produce targeted and controlled drug delivery technologies [125]. An evaluation of NS for enhancing the anticancer activity of CUR has been reported. The NS based formulation exhibited greater cytotoxicity against MCF-7 cells while exhibiting sustained release and increased solubilization of CUR, with cells incubated in blank culture medium used as a control for cell viability assessments [126,127]. Similar outcomes were observed when CUR was encapsulated in a nano-sponge technology and their toxicity assessed in a MCF-7 cell line [128].

Liposomes are bi-layered nano drug delivery systems that have been used to improve the delivery of CUR for anti- cancer therapy. Saengkrit et al. [129] demonstrated improved CUR delivery in a cervical cancer model and the results suggest CUR containing nano-liposomes had increased anti-cancer activity than free CUR curcumin when compared at the same concentration. In addition, apoptosis assays indicated that inclusion of a positive charge on the surface of the liposome by addition of didodecyl dimethylammonium bromide (DDAB) improved the anti-cancer activity of CUR.

Similarly, synthetic cholesterol based cationic lipids were shown to be effective as delivery systems whilst enhancing stability, cellular uptake and cytotoxicity of CUR, with cytotoxicity of CUR encapsulated liposomes being two–eight-times greater than that of free CUR [130].

A unique CUR containing cyclodextrin liposome enhanced the aqueous solubility of CUR and resulted in greater entrapment of CUR in the aqueous core of the liposomes. This novel drug delivery system demonstrated that a lower concentration of CUR was required to achieve a desired therapeutic effect when treating osteosarcoma with human mesenchymal stem cells used as the control for osteosarcoma and control tumours treated with empty liposomes [131].

Niosomes are similar to liposomes in that they are bilayered and capable of entrapping hydrophilic and hydrophobic moieties. However, they are synthesised using non-ionic surfactants rather than phospholipids [132,133]. A novel CUR-loaded niosome formulation was effectively used to deliver CUR for the treatment of ovarian cancer [134]. The niosome formulation demonstrated that the technology ensured biochemically stable protection and high entrapment efficiency for CUR in addition to displaying enhanced cellular uptake, greater cytotoxic activity, efficient cell cycle arrest and apoptotic rate in the ovarian cancer A2780 cell line thereby exhibiting potential as a strategy for the treatment of ovarian cancer [134].

Sahab-Negah et al. [135] prepared CUR niosomes to evaluate their potential as an effective treatment approach using glioblastoma stem-like cells (GSC). The results revealed that the niosome formulation containing CUR exhibited a significant increase in anti-tumour activity. Furthermore, the niosomes were more efficient at targeting viability, proliferation and migration of GSC than free CUR by multiple molecular mechanisms, including induction of cell cycle arrest, apoptosis, and ROS generation with un-treated GCS used as a control group.

It has been reported that the loss of phosphatase and tensin homolog (PTEN) protein frequently results in prostate cancer in humans [136]. In a PTEN knockout mouse model, CUR in combination with resveratrol in liposomes resulted in increased concentrations of CUR in tumour cells. This increase in bioavailability of CUR cured prostate cancer by induction of apoptosis and reduced proliferation of prostatic adenocarcinoma cells in vivo. Mice with histologically normal prostates were dosed with empty liposomes and used as the control group in these studies [137].

### 3.2. Anti-Oxidant Activity

CUR is a natural component of the human diet that has antioxidant activity and has been used for the prophylaxis and treatment of disease normally associated with or precipitated by oxidative stress [138]. To evaluate the impact of nano-encapsulation on the antioxidant activity of CUR, lipid-core nano-capsules containing CUR were prepared using precipitation of a preformed polymers and it was established that the in vitro antioxidant activity of CUR against OH radicals was enhanced when using nano-encapsulation. Antioxidant activity was determined using an artificial and controlled generation of hydroxyl radical by decomposition of H_2_O_2_ with an ethanolic solution used as the control. In addition, the nano-capsules exhibited controlled release CUR profiles [138]. To preserve the antioxidant activity of CUR by preventing photodegradation following topical application of cosmetics the compound was encapsulated using ethyl cellulose and/or methyl cellulose nano-particles [139]. Following exposure to the sun it was revealed that nano-encapsulation enhanced the photostability of CUR when compared to un-encapsulated CUR which degraded completely. Untreated skin samples served as the control, in particular, untreated irradiated skin samples served as a positive control for radical formation while non-irradiated samples were used as a negative control. Furthermore, radical protection factor evaluation exhibited similar antioxidant activity when encapsulated and un-encapsulated CUR were tested indicating that antioxidant activity had been maintained [139]. The encapsulation of CUR using medium chain triglyceride oil droplet nano-emulsions prepared by ultrasonification using whey protein concentrate 70 and Tween 80 as emulsifiers has been reported [140]. Interestingly, total antioxidant activity of encapsulated CUR was slightly lower than that of free CUR viz., 3.33 ± 0.02 versus 3.53 ± 0.11 02 μM Trolox/mg of CUR, respectively. It was postulated that encapsulation not only protects CUR from degradation but may likely preserve antioxidant activity, thereby enhancing the pharmacological properties and activity of the compound. To investigate the effect of encapsulation on the anti-oxidant activity of CUR, the polyphenol was nano-encapsulated using dichloromethane as a solvent and ultrasonication for the manufacture of poly(l-lactic acid) and Eudragit S100 as nano-capsules. Following antioxidant activity testing prior to and following encapsulation it was concluded that encapsulation had no detrimental impact on the antioxidant activity of CUR [141]. 

The pharmacokinetics of CUR encapsulated in liposomes of approximately 263 nm diameter was investigated. The pharmacokinetics of CUR-loaded liposomes in Sprague-Dawley (SD) rats was compared to the pharmacokinetics of a mixture of CUR and lecithin and free CUR (control group). The liposomes formulation exhibited higher bioavailability, a faster rate and extent of absorption when compared to the other administered forms. Oral administration of the liposome formulation resulted in a higher maximum plasma concentration (C_max_) with a shorter time to reach maximum concentration (T_max_). Furthermore, the use of the oral route of liposome administration revealed better antioxidant activity in plasma [142].

Liposomes of varying size were manufactured to encapsulate CUR/curcuminoids and were subsequently characterised. The antioxidant activity of pure CUR and CUR in the liposome formulation were compared using 1,1-diphenyl-2-picrylhydrazyl radical scavenging and superoxide dismutase activity. The liposome formulations exhibited significantly greater anti-oxidant activity than pure CUR. Furthermore, antioxidant activity of CUR/ curcuminoids was compared to that of vitamin C, lipoic acid, and NAME (a known iNOS inhibitor) with a pure DPPH solution used as the control [143].

The anti-oxidant activity of CUR containing niosomes co-formulated with hyaluronan was investigated and the results suggested that the niosome formulation exhibited superior anti-oxidant activity when compared to CUR suspensions of the same strength [144].

Similar results were observed in experiments where CUR was co-encapsulated with other antioxidants to produce a greater antioxidant effect [145,146]. The effect of co-encapsulation of CUR with other antioxidants suggested that while niosome formulations increased the antioxidant activity of CUR, additional synergistic antioxidant effects were possible [145]. In an identical study the effect of co-encapsulating CUR with α-tocopherol or with resveratrol was evaluated and in both instances the antioxidant activity was compared to that using niosomes containing the individual drug and free drug. In both instances the co-encapsulated drugs exhibited superior antioxidant activity than any of the individual cases with blank DPPH solutions and empty niosomes used as study controls [146].

Nano-sponges containing CUR were synthesised with the intention of assessing their ability to enhance delivery of CUR and this drug delivery option was thought to be capable of delivering the otherwise insoluble CUR using an aqueous based technology [147]. The results suggest that the nano-sponge formulation increased the antioxidant activity of CUR. Zein/carboxymethyl dextrin nanoparticles were fabricated to deliver CUR and the antioxidant activity of loaded nanocarriers determined by monitoring the scavenging activity of CUR for the DPPH radical. These data were compared to the scavenging activity of free CUR in water (control). Zein/ carboxymethyl dextrin CUR-loaded particles exhibited a scavenging ability of 68.25% compared to 19.56% for the control which was attributed better dispersion of encapsulated hydrophobic CUR in/on the hydrophilic surface of zein/carboxymethyl dextrin in water, thereby increasing contact between CUR and free radicals. These findings further illustrate that encapsulation of CUR in zein/carboxymethyl dextrin nanocarriers was effective in enhancing the antioxidant capacity of the molecule [148].

### 3.3. Anti-Viral Activity

CUR has been reported to possess inhibitory effects against viruses, including the influenza A, herpes simplex type 1, Zika and SARS-CoV viruses through interference of critical steps in the lifecycle [58,149,150]. CUR-loaded apotransferrin nanoparticles prepared by sol-oil chemistry were able to enter T-cells through transferrin-mediated endocytosis, exhibited sustained release properties and were approximately 50% less toxic than pure CUR and showed higher anti-HIV activity compared to pure CUR in vitro. In this study, the authors defined 0% inhibition as the absence of viral replication in untreated samples while using azidothymidine as a positive control [151]. Enhanced synergistic anti-viral effects of CUR and silver nanoparticles in a tissue culture infectious dose assay study showed that CUR modified silver nanoparticles were highly efficient at inhibiting respiratory syncytial virus (RSV) with a resulting and significant decrease in the viral titer of RSV in comparison to that observed with silver nanoparticles capped with citric acid. In addition, little to no toxicity was observed in host cells following treatment with CUR modified silver nanoparticles. Furthermore, the study protocol included negative and positive controls, viz. blank cells and cells infected with RSV without nanoparticles, respectively [152]. The effect of CUR containing nano-emulsions, prepared by solubilization of CUR in the oil phase followed by aqueous phase homogenization with a hydrophilic emulsifier, on vulvar intraepithelial neoplasia which is associated with human papillomavirus (HPV) infection, was investigated. Good biocompatibility of the CUR containing nanoemulsions was observed and cytotoxicity assay data revealed that in comparison to free CUR, the CUR-loaded nano-emulsions improved cell viability of up to 80% in comparison to the approximately 15% observed for the pure drug. These results were obtained with the use of DMEM and DMEM+ photodynamic therapy as controls during the organotypic culture studies. Furthermore, intracellular uptake assays revealed that CUR was more efficiently internalised into cells following nano-emulsification and different HPV E6 variant expression did not affect drug uptake [153].

### 3.4. Anti-Bacterial Activity

Extensive research has revealed that bacterial infections are among the top causes of infectious diseases worldwide. Following preparation of CUR containing nano-particles using a process based on a wet-milling technique, the nano-carriers were found to be much more effective than pure CUR against *Staphylococcus aureus*, *Bacillus subtilis*, *Escherichia coli*, *Pseudomonas aeruginosa*, *Penicillium notatum* and *Aspergillus niger.* plates containing pure DMSO were used as the control in zone inhibition experiments [154].

Zinc oxide-grafted CUR containing nano-composites with different morphologies were manufactured and their anti-bacterial activity evaluated [155]. Despite all zinc oxide and zinc-grafted CUR containing nano-particles exhibiting anti-bacterial activity, spherical ZnO–CUR and rod-shaped ZnO–CUR nano-particles showed the most favorable antimicrobial activity against the bacterial strains tested with distilled water and gentamicin used as the negative and positive controls, respectively. In another study, micelles comprised of poly(lactic-*co*-glycolic acid) and dextran were prepared and functionalised with CUR and the activity of the particles against *Pseudomonas* spp. and biofilms investigated. The micelle product had a greater ability to penetrate biofilms resulting in superior antibacterial activity when compared to pure CUR. In addition, the functionalised carriers disrupted mature biofilms and exerted anti-bacterial activity against biofilm-embedded cells [156]. For the antibacterial and anti-biofilm assays untreated bacterial culture medium were used as the control. 

Pandit et al. [157] synthesised nanoparticles containing CUR using ultrasonication. Following successful nano-encapsulation of CUR the in vitro antibacterial activity was evaluated against *Escherichia coli*, *Staphylococcus aureus* and *Pseudomonas aeruginosa*. The CUR-loaded nanocarriers were found to be more effective against the bacterial species in comparison to unencapsulated CUR and a CUR free cream (control), and were thus considered promising formulations for the treatment of bacterial infections and wound healing in humans.

With the aim of implementing a novel strategy to remove different bacterial pathogens from hospital wastewater an eco-friendly CUR-loaded nano-structured lipid carriers was prepared using hot high-speed homogenization [158]. The in vitro antibacterial activity of pure CUR and the nano-carriers evaluated using a turbidity assay in Luria–Bertani media and standard and wild-type bacteria strains. The results of these studies suggested that the CUR nano-particles (0.125 μM) in Mueller–Hinton agar media significantly reduced the number of wild-type bacterial strains in autoclaved wastewater at 37 °C and the microbial total count at 25 °C in original hospital wastewater when compared with pure CUR.

A high throughput screening study conducted using 28 candidate compounds tested using anti-solvent precipitation produced CUR nano-particles of target size, particle size distribution and ζ-potential. The ligand-protected CUR nano-particles exhibited anti-microbial activity against Gram-negative *Escherichia coli* ATCC 25922 with MIC values of 400–500 µM and the effect of stabilizing ligands on the anti-microbial activity of the particles required further investigation. These studies included *E. coli* with nutrient medium with or without stabilizing ligands as positive controls [159].

Kianvash et al., [160] manufactured CUR loaded liposomes coated in polyethylene glycol for the treatment of burns. The liposome-based CUR formulation were found to have similar anti-bacterial activity to silver sulfadiazine with empty liposomes as the control, and it was concluded that low dose CUR nano-liposome formulations improved injury and infection of burns efficiently and may be a successful approach for burn therapy.

### 3.5. Anti-Inflammatory Activity

The anti-inflammatory activity of CUR has mainly been attributed to inhibition of activity of enzymes such as cyclooxygenase, lipo-oxygenase, phospholipase A2, and other mechanisms of action [161,162]. Self-assembly of chitosan and fucoidan was used to encapsulate CUR to form multi-stimuli-responsive nano-carriers. The carriers were delivered to the brain of mice, intranasally, and showed improved delivery, distribution and accumulation of CUR in inflamed brain lesions while using untreated healthy mice as the control animals [163]. High-speed and high-pressure homogenization was used to prepare CUR-loaded oil-in-water nano-emulsions prepared with medium chain triacylglycerides and Tween 20. Enhanced anti-inflammatory activity of CUR loaded nano-emulsions was observed in a 12-*O*-tetradecanoylphorbol-13-acetate (TPA) -induced mouse ear inflammation model while pure CUR solution and drug free emulsions (control) showed little and no inhibitory effect of TPA-induced edema in mouse ears, respectively [164]. CUR-loaded nano-structured lipid carriers (CUR-NLC) were prepared and loaded into a gel to produce a CUR-NLC-thermo-sensitive in situ gels for topical delivery and in vivo studies revealed that the CUR-NLC-Gel formulation exhibited significant anti-inflammatory effects in a xylene-induced ear edema mouse model when compared to treatment with a blank gel matrix (control group). The authors also applied 1 mg indomethacin/ear to the positive control group animals [165]. To evaluate the in vivo anti-inflammatory and wound healing efficacy of CUR TPA was applied to the shaved backs of female CD-1 mice. The results of this study showed that animals treated with CUR-loaded hyalurosomes, in comparison to the controls viz., saline and empty hyalurosome-treatment, exhibited minimal signs of inflammation, edema and redness, which was attributed to the increased ability of encapsulated CUR to downregulate inflammatory reactions compared to that observed in the other treatment groups. In addition, these findings were also compared to healthy untreated skin [166]. Merrell et al. [167] investigated the potential of poly (caprolactone) nano-fibers as a delivery vehicle for CUR in wound healing applications. The CUR-loaded nano-fibers were found to diminish inflammatory induction by reducing the levels of interleukin-6 release from mouse monocyte-macrophages located on the fibers following stimulation by *Escherichia coli*-derived lipopolysaccharide.

The ability of CUR loaded liposomes in reducing inflammation was demonstrated in a study in which liposomal CUR was used to target cellular delivery to renal tubular epithelial cells (TEC) and antigen presenting cells (AAPC) and compared to empty liposome (vehicle control) in vivo. Protection from ischaemic reperfusion injury (IRI) was mediated by NF-κB. Liposomal endocytosis of CUR by both cell compartments is advantageous in IR as the renal TEC are the main site of hypoxic damage and oxidative stress following IR injury, and resident APC mediates the innate immune response to parenchymal injury [168].

The anti-inflammatory activity of liposome containing CUR was assessed and compared against raw CUR by measuring the inhibition of lipopolysaccharide-induced nitric oxide, interleukin-1β and tumour necrosis factor production in macrophage RAW 264.7 cells. In these studies, the anti-inflammatory activity of the liposome-based formulation was superior when compared to free CUR while using 0.2% dimethyl sulfoxide medium as the control [143].

## 4. Future Considerations of Encapsulation

The available evidence suggests that the therapeutic benefits derived from using encapsulated curcumin may be significant and future research is required to focus efforts on the enhancement of oral absorption and the bioavailability of curcumin as this will facilitate an understanding of this therapeutic agent for clinical treatment of human disease [169]. The extensive anti-viral effects of curcumin against pathogens suggest that the compound may be an effective anti-viral drug candidate which may be developed further and highlights the potential of natural resources as a source of compounds which would be useful against viruses [169]. Future research must focus on the manner in which curcumin and derivatives can be used as anti-viral agents. Nano-technology based formulations containing curcumin have been tested against a range of bacteria and fungi [170]. Future research must necessarily focus on the analysis of nano-encapsulation by establishing reasonable basis for making comparisons. The evidence provided has discussed a range of strategies for increasing the solubility and absorption of curcumin however, future research must analyze features of nano- and micro-encapsulation, which can be enhanced taking cognizance of cost, stability, industrial production, scale up and regulatory requirements [171]. The analysis of micro-encapsulation of curcumin revealed that micro-particles exhibit efficient muco- and bio-adhesive properties and efficient bio-adhesion results in improved bioavailability of curcumin, ensuring higher plasma concentrations of the drug may be achieved for a longer period [171]. Future research is required to investigate the impact of encapsulation of curcumin micro-particles using different human model pathologies.

## 5. Conclusions 

Curcuminoids support gene expression and epigenetic mechanisms by exhibiting biological activity. However, the extremely low solubility of CUR in aqueous buffer systems, poor bioavailability, rapid metabolism and instability in body fluids has minimised the clinical potential and therapeutic use of curcumin. The use of encapsulation processes to convert small core-material particles into a capsule by encasement in protective shells is a functional approach to modulate the properties of products based on composition and material used. The encapsulated product is defined by the size of the particle and nano-encapsulated particles range in diameter from 10 to 100 nm, whereas micro-encapsulated particles have diameters ranging between 3 and 800 μm. It is evident that CUR has significant therapeutic benefits including anti-cancer activity which has contribute to a significant improvement in melanoma, breast, lung, gastro-intestinal and genito-urinary cancers. Nano-encapsulated curcuminoids exhibit bioactive properties, exert cytotoxic and antioxidant effects, and act on endogenous and cholinergic antioxidant systems. CUR blocks the HCV entry pathway in hepatoma cells, inhibits MRSA growth, enhances wound healing, and reduces bacterial load. It is evident that CUR is a remarkable molecule requiring future research to focus investigations of encapsulation and testing in different models of human pathology. Notwithstanding, the literature and research cited in this review requires in depth interrogation to establish alignment of experimental procedures and use of study controls. Furthermore, the use of physiologically relevant doses of CUR during experimental studies and availability of toxicity data in humans is necessary, prior to considering dosage form and process design activities for translation into clinical settings.

## Figures and Tables

**Table 1:** Summary of methods used to encapsulate bioactive compounds and materials [15,120].

	Physical Method	Chemical Method	Physico-Chemical Methods
Basic Principle	The bioactive material is coated and dried to produce micro- or nano-particles.	Integration of polymerization to entrap bioactive compounds. Useful for encapsulating solid cores of small size and liquids.	The micro- nano-particle wall is formed using synthetic or natural preformed polymers.
Examples	Supercritical fluid mediated process Freeze drying Crystallization Multi-orifice centrifugation Extrusion Fluid bed coating Spray cooling Spray chilling Spray drying	Interfacial cross-linking Interfacial polymerization In situ polymerization Interfacial polycondensation	Liposome entrapment Simple or complex coacervation Solvent evaporation Ionic gelation Hot melt coating Emulsification

## Data Availability

Not applicable.
